# Beyond Binge Eating: The Impact of Implicit Biases in Healthcare on Youth with Disordered Eating and Obesity

**DOI:** 10.3390/nu15081861

**Published:** 2023-04-13

**Authors:** Karyn J. Roberts, Eileen Chaves

**Affiliations:** 1 College of Nursing, University of Wisconsin-Milwaukee, 1921 E Hartford Avenue, Milwaukee, WI 53211, USA; 2 Department of Pediatrics, Feinberg School of Medicine, Northwestern University, Chicago, IL 60611, USA; 3 Nationwide Children’s Hospital, The Ohio State University, 700 Children’s Drive, Jwest 3rd Floor Columbus, Columbus, OH 43205, USA

**Keywords:** eating disorders, disordered eating behaviors, stigma, obesity, intersectionality, pediatrics, race, gender diverse

## Abstract

(1) Background: Obesity and eating disorders (ED) can coexist resulting in worse health outcomes. Youth with ED are more likely to have obesity relative to peers with a healthy weight. Pediatric providers deliver first-line care to children and youth of all sizes and body shapes from infancy to adolescents. As healthcare providers (HCPs), we bring biases into our practice. Learning to recognize and address these biases is needed to provide the best care for youth with obesity. (2) Purpose: This paper aims to summarize the literature regarding the prevalence of ED beyond binge eating in youth with obesity and discuss how the intersection of weight, gender, and racial biases impact the assessment, diagnosis, and treatment of ED. We provide recommendations for practice and considerations for research and policy. (3) Conclusions: The assessment and treatment of ED and disordered eating behaviors (DEBs) in youth with obesity is complex and requires a holistic approach. This approach begins with identifying and understanding how one’s implicit biases impact care. Providing care from a patient-centers lens, which considers how the intersection of multiple stigmatized identities increases the risk for DEBs in youth with obesity may improve long-term health outcomes.

## 1. Introduction

Eating disorders (ED) disrupt eating or eating-related behaviors that alter the consumption or absorption of food and impair physical and psychological health and functioning [1,2]. Obesity in children/adolescents is defined as having a body mass index (BMI) ≥ 95th percentile for age and sex and is increasing in prevalence worldwide [3,4]. Comorbidities of ED and obesity include both physical and psychological sequelae which negatively impact long-term physical and psychological health and quality of life [5,6,7,8,9]. Additionally, the etiology of ED and obesity may overlap and can include genetic predisposition, and environmental, cognitive, and behavioral risk factors [2,10]. Obesity and ED can coexist resulting in worse health outcomes [2,11]. Lifetime prevalence of ED in adults in the general population occurs in 2.22% of males and 4.9% of females and is most prevalent in those with the highest levels of obesity [11]. In children and adolescents, the lifetime prevalence of ED ranges from 1.4–6.2% depending on age and diagnosis [12,13,14,15]. Adolescents with ED are more likely to have obesity relative to those with healthy weight [2,15,16]. To our knowledge there is no data available regarding the prevalence of ED in youth with obesity, nor have any longitudinal analyses been conducted of risk factors for the onset of ED in this population [2].

In pediatric practice, the assessment of ED is often incomplete or absent, and, therefore, ED is underdiagnosed, particularly in youth with obesity. Multiple factors account for the omission of ED assessment, including lack of training for assessment and diagnosis of ED and poor understanding of ED risk in youth due to gender and racial stereotypes [2,17,18]. Additionally, there is a low threshold to assess for ED in youth with obesity beyond binge eating disorder (BED) by providers of pediatric care due to implicit/explicit weight bias and stigma [2]. HCPs often wrongly assume that if a youth with obesity has an ED this ED is BED, overlooking the range of disordered eating behaviors that could be present, such as restricting or compensatory behaviors. DEBs describe a range of disordered eating, including clinical diagnoses and subclinical presentations of ED. Providers of pediatric weight management have reported missed diagnoses of ED resulting in poor outcomes and difficulty following weight management recommendations [2]. This has led to increased vigilance in assessing for and treating ED and DEBs in youth with obesity in pediatric weight management programs [2].

Pediatric providers deliver first-line care to children and youth of all sizes and body shapes from infancy to adolescents. Providers need tools and strategies to effectively assess and treat ED. To provide the best care to youth with obesity, awareness and understanding of the range of DEBs are needed. As HCPs, we bring biases into our practice. Learning to recognize and address these biases is needed to provide the best care for youth with obesity. This paper aims to summarize the relevant literature regarding the prevalence of ED (beyond BED) in youth with obesity and discuss how the intersection of weight, gender, and racial biases impact the assessment, diagnosis, and treatment of ED. We provide recommendations for practice and considerations for research and policy.

## 2. Eating Disorders and Disordered Eating

The terms ‘ED’ and ‘disordered eating’ are often used interchangeably, though these concepts differ in the frequency and severity of the behaviors they represent. Both concepts involve eating behaviors that interfere with an individual’s quality of life and the associated cognitions that present with these behaviors [19]. For the purposes of this manuscript, the term disordered eating behaviors (DEBs) will be used for consistency.

When diagnosing an ED, the frequency and severity of an individual’s atypical eating pattern determine if the pattern aligns with subclinical ED symptoms (i.e., disordered eating) or a diagnosable ED. For example, when diagnosing bulimia nervosa (BN), an individual must have recurrent episodes of binge eating (i.e., consuming a large amount of food within a specified time) and experience a lack of control over their eating. Additionally, the individual uses compensatory behavior after the binge episode to prevent weight gain such as self-induced vomiting, misuse of laxatives/diuretics, excessive exercise, or fasting. To meet diagnostic criteria for BN, these behaviors must occur at least once a week for three months accompanied by a negative self-evaluation of the individual’s body shape and size [1].

Adolescent girls are at the highest risk of developing an ED [20]. While the lifetime prevalence of ED among adolescent girls is 0.7% for anorexia nervosa (AN) and 1.2% for BN, the prevalence of adolescent girls with subclinical ED symptoms is estimated to be 44% [21]. It is important for primary care providers to have a baseline knowledge of subclinical ED symptomology and awareness of DEBs. Without this knowledge and heightened vigilance, missed assessments and diagnoses of DEBs are common. In youth with obesity, subclinical ED behaviors often present as dieting, fasting, or other weight-control behaviors. A provider may initially assess these behaviors as adherence to weight management recommendations. However, with further assessment, these behaviors and their associated cognitions may provide a more complete understanding of an individual’s negative thoughts and feelings about their body, as well as maladaptive behaviors used to induce weight loss. While these behaviors may not occur with the frequency or severity needed to diagnose an ED, they are problematic and are associated with mood and anxiety disorders as well as complicating treatment for obesity [21]. For a comparison between ED diagnoses and the subclinical presentations, see Table 1.

## 3. Intersectionality

It is necessary to consider the ways an individual’s various identities interact to advantage or disadvantage their health outcomes when providing health care. The term intersectionality refers to a way of understanding an individual’s multiple, interconnected social categories (i.e., race, gender identity, and sexual orientation) [23]. These categories interact at individual and structural levels and impact health outcomes [24]. Depending on how a provider engages with a patient, the category can confer advantages or disadvantages to the patient’s health outcomes. For example, when providing care to youth with obesity, a Latine adolescent female may be categorized either as “female”, “Latine”, or “having obesity”, two of the above, or all the above. Each of these classifications provides a level of advantage or disadvantage to the individual. For individuals with multiple devalued social categories, the cumulative disadvantages may outweigh the risk of one individual social category alone, though the impact is not simply additive [25,26,27].

In a study examining the intersection of race, gender, and weight stigma in Asian, Black, Hispanic, and White men and women (*n* = 3088), Himmelstein et al., (2017) reported on experiences of stigma and coping responses [28]. They noted that despite a higher prevalence of obesity in Black and Hispanics, in studies examining weight stigma race is often only a control variable [28]. This methodological approach misses protective or detrimental health factors by failing to systematically examine race and gender in weight stigma [28]. Overall, they found weight stigma is not just a White woman’s issue but may be experienced equally across race and gender [28]. However, internalized weight bias (i.e., weight stigma directed at oneself) was less among Blacks and Hispanics compared to White individuals [28]. In coping with stigma, Hispanic women and Black men were more likely to engage in disordered eating compared to white women and white men respectively [28]. Whereas Black women were less likely to cope with the stigma by disordered eating than White women [28]. Though this study was in adults, it is an example of how in the care of youth with obesity, consideration is needed for how their various identities may elevate their risk for DEBs and worsen into adulthood without intervention [29].

## 4. Weight Stigma

Weight stigma is the social devaluation and denigration of a person due to their excess body weight and can lead to overt discrimination [30]. Weight-based stereotypes are generalizations that individuals with obesity are to blame for their weight due to laziness, lack of self-discipline, and noncompliance with medical treatment [30]. Children with obesity as young as 3–5 years old have been negatively stereotyped due to their weight [31]. These societal stereotypes fuel implicit and explicit biases against persons with obesity as well as weight stigma.

Weight-based stigmatization and mistreatment are especially prevalent in youth with obesity regardless of their sociodemographic characteristics [32,33,34,35]. In the US, 13–32% of youth report being discriminated against, and 25–50% of all youth report having been bullied due to their weight [33,35,36,37,38,39,40]. Ethnically diverse adolescents in the US (*n*= 162,034) were more likely to be bullied for their weight than for their sex, race/ethnicity, sexual orientation, or disability [33,41]. Work across countries has reported similar findings. Youth with overweight and obesity are more likely and more frequently teased and bullied due to their higher weight compared to peers with “normal” weight [33,42,43]. Adolescents, teachers, and adults across countries consistently report that body weight is the primary reason youth are teased and bullied [44,45,46,47].

Manifestations of weight stigma include weight-based teasing and bullying, physical acts of violence, social exclusion, name calling, and blaming [33,46,48]. Weight-based victimization (WBV) is defined as bullying or teasing resulting from an individual’s weight [30]. Weight stigma and WBV contribute to an increased likelihood of adverse health behaviors, including DEBs, unhealthy weight-control behaviors, and lack of physical activity [49,50,51,52].

Sources of stigma include peers, family, teachers, society, media, healthcare providers, and individuals themselves [33,53,54,55,56,57,58]. Internalized weight bias occurs when an individual directs stigma and negative stereotypes regarding weight-biased beliefs and attitudes toward themselves [59]. Primary care providers may frame weight as an urgent health risk without an awareness of their own implicit weight bias and inadvertently do harm. These experiences of weight stigma in persons with obesity result in stress and mistrust of healthcare providers [60]. Additionally, these experiences impact patient engagement, motivation, and adherence; prevent timely access to care, and reduce the quality of care provided; and can lead to DEBs, additional weight gain, poor outcomes, and withdrawal from care [32,60,61,62].

## 5. Sexual Minority and Gender-Diverse Youth

Sexual-minority youth (i.e., lesbian, gay, and bisexual) have disproportionate rates of DEBs compared to their heterosexual peers [9,63,64,65,66]. Similarly, sexual-minority females have a higher body-mass index (BMI), and sexual-minority males have a lower BMI than their same-gender heterosexual peers [67,68]. Watson, et al., (2017) reported DEBs have reduced over time in same- and both-sex partnered youth compared to opposite-sex partnered youth, while the rates of DEBs among same-sex partnered females have increased [65]. Analysis of Youth Risk Behavior Survey data shows lesbian, gay, and bisexual high school students have significantly higher rates of DEBs focused on weight control than their heterosexual peers [9,69,70].

Prevalence rates of ED in gender-diverse youth vary widely from 2–18% [71]. Gender nonbinary individuals have a 2–4 times greater risk of DEBs than their cisgender peers [72]. Transgender youth may be at higher risk due to body dysmorphia and dissatisfaction, which may be relieved by gender reassignment surgery [9,73,74]. In a survey of 289,024 college students from 223 U.S. universities, transgender individuals had the highest rates of self-reported DEBs compared with all cisgender groups [75]. Nearly 16% of transgender students reported being diagnosed with an ED in the past year compared to 1.85% of cisgender heterosexual women [75]. Additionally, 13.5–15% of transgender students reported DEBs in the past month compared to 3.71–4.29% of cisgender heterosexual women [75]. The literature is evolving in this area, however, there have been no studies to date that definitively determine the role of gender identity in the etiology of DEBs in gender-diverse or transgender populations.

A study with LGBTQ gender-diverse individuals with obesity seeking primary care, described “Fat Broken Arm Syndrome” [76]. Study participants perceived providers as attributing all health concerns to weight and offering weight loss as a solution regardless of the presenting concern [76]. Patients experienced heightened stigma due to their sexual orientation, gender identity, and bigger body [76]. Paine (2022) concluded healthcare professionals often apply a “moral-medical framing of fat”, which blames patients for individual choices and communicates the urgent health risk of obesity, regardless of the presenting complaint [76]. This is an example of how multiple stigmatized identities compound the stress on youth with obesity and increase their risk for DEBs.

Recognizing that the experiences of transgender youth may be different depending on how they present for care is important to consider. For gender-diverse youth with obesity, there is a complex relationship between their physical appearance, body image, eating patterns, gender expression, mood, and the ability to exercise [77]. It is important for primary care providers to have an awareness of the complexity of this relationship, as it may increase the risk of DEBs and obesity. A pattern of eating, either through restriction or overeating, may be used to foster an individual’s preferred gender expression through weight loss or weight gain and may improve with gender-affirming care and treatment [71].

## 6. Race

Previous research on the rates of ED has primarily been done in White, non-Hispanic, and cisgender populations [68]. Obesity disproportionately affects minoritized and under-resourced communities with Black (12.8%) and Hispanic (12.4%) youth nine times more likely than Asian youth (1.4%) and 2.5 times more likely than White youth (5%) to have severe obesity [3]. In a large (*n* = 13,200) U.S. nationally-representative cohort of youth 11–21 years of age, Katz-Wise (2014) described an increase in age-specific BMI in males and females across sexual orientation and race/ethnicity [68].

Weight-based teasing and bullying resulting from weight stigma affects children of all races and ethnicities. Several studies have shown that children and adolescents of some race and ethnic groups (Hispanic and White girls) experience WBV from either peers or family members more often than youth of other ethnicities, though other studies report that WBV affects all youth equally [78,79]. Youth who experience WBV can develop maladaptive eating patterns, resulting in ED and a decreased quality of life.

ED and DEBs have historically been thought to primarily affect “skinny, white, affluent girls” (SWAG stereotype) [18], resulting in decreased screening, missed diagnoses, and treatment of Black Indigenous People of Color (BIPOC), males, and individuals from lower socioeconomic backgrounds with overweight or obesity. Gordon et al., (2006), presented case scenarios to clinicians of individuals presenting with DEBS [80]. Different groups of clinicians were presented with a same case scenario of a 16-year-old female named Mary [80]. Mary’s clinical presentation of DEBs was the same for each clinician group though her race (Black, White, or Latine) was different in each group. Clinicians were asked to decide if Mary had an ED and if they would refer her for treatment. When Mary presented as White or Latine she was more often diagnosed and referred for treatment, than when she presented as Black, in most cases providers failing to diagnose an ED [80].

Implicit biases by HCP informed by research, cultural norms, and stigma have reinforced the false narrative that BIPOC youth with obesity only display a narrow subset of DEBs, usually binge eating. BIPOC youth with obesity may display a wide range of DEBs beyond binge eating, but they are at higher risk of missed screening, diagnoses, and treatment for DEBs. All these factors contribute to an increased risk of poor health outcomes in BIPOC youth with obesity.

## 7. Recommendations for Practice

### Assessment

It is important for primary care providers to have a high index of suspicion for ED and DEBs in youth with obesity, particularly among adolescents [65,73]. This begins with a consideration of one’s implicit biases in providing care. Acknowledging how implicit biases may affect HCP’s approach, assessment, and treatment of this vulnerable population is necessary for holistic care. It should be assumed that youth with obesity are experiencing stigma from multiple sources in their daily life, including in their own home. Understanding the complex intersection of the multiple victimized, marginalized, and stigmatized identities (e.g., gender, race) of youth with obesity is vital. Providers should use this understanding in developing goals for treatment that should include reducing the negative impact of these social complications [46,77,81]. For example, providers can refer patients who have anxiety or depression from teasing, bullying, or WBV episodes to a mental health provider. HCPS can provide referrals to community resources, gyms, or programs that are inclusive of various body sizes, and which encourage participation in intentionally inclusive activities, i.e., Girls on the Run, etc.

Assessments and interventions for obesity should focus on healthy body image and healthy approaches. Providing screening for DEBs in all youth with obesity should be presented in nonjudgmental ways. Table 2 provides specific language to use in the assessment of DEBs. This approach begins with being aware of and assessing one’s own implicit biases related to individuals in larger bodies. Screening for DEBs should be adopted into practice, much as how screening for mental health and suicidal ideation has become the standard of care. This screening should begin as part of routine care for children beginning in prepuberty [9]. It should be clear to youth and their caregivers that these questions are asked of all patients. There are many validated screening tools available for ED [82,83,84,85]. However, current diagnostic tools may not be adequate for some minoritized populations, i.e., transgender, etc., and improved and validated measures are needed [71].

Pediatric providers should advocate for and provide weight-neutral and inclusive care to youth with obesity. Weight is not proxy for health and should not be discussed with patients and family members unless permission is given and/or concern about weight is the reason for the patient’s visit. Additional considerations should be given to the need to physically weigh patients. If it is medically necessary, weights should be taken in private spaces, and HCPs should ensure furniture and equipment is adequate for persons in larger bodies. The use of first-person language when discussing or documenting about a patient (“child with obesity” rather than “obese child”) and avoiding words like “fat”, “large” or “obese” and use of “weight” or “BMI” in speaking with youth and their families is critical [86,87]. Education and skill are also needed to provide gender-affirming care for sexual minority, gender-diverse, and transgender youth with obesity.

**Table 2:** Assessing Disordered Eating Behaviors in Adolescents [88] *.

Goal	Scripting
Start a conversation about eating habits	Would it be okay if we discussed your eating habits? May we discuss how you typically eat? Have you done anything in the past 6 months to change your weight?
Assess motivation to change eating habits	On a scale of 0–10, how important is it for you to change your eating? What would make it more important? What do you like about the way you eat? What do you dislike? How would your life be different if you did not need to spend so much time thinking about your eating?
Determine the antecedents and consequences of disordered eating patterns	Do you ever feel that you lose control over the way you eat? Do you ever intentionally restrict your food intake, or skip meals when you are hungry? Do you ever vomit/purge after you feel you have lost control with your eating? How often does that happen? Sometimes people binge, purge, or restrict their eating when they feel emotional (sad, stressed, bored, worried, etc.) Do any of these situations apply to you? How does eating or not eating affect your ability to function during the day?

* Assessment should occur in privacy without caregiver presence if possible.

## 8. Recommendations for Research and Policy

More research is needed to understand the complexities of ED/DEBs in youth with obesity. Areas to consider include the interaction of genetic and environmental risk factors, particularly concerning eating behaviors in family units. How do cultural and family attitudes and behaviors surrounding food, eating, and expression of emotions influence DEBs? What influence does genetics have on satiety and eating behavior? Research into the barriers and facilitators in screening for ED/DEB by HCPs could bridge the gap between research and best practice and improve early diagnosis and treatment for ED for those youth at the highest risk. Research is needed on the intersection of obesity and ED in the contexts of sexual-minority and gender-diverse youth. Interventional research which is codesigned with traditionally underrepresented and minoritized youth and their families, i.e., BIPOC, LGBTQ, is needed to create best practices, improve holistic health, quality of life, and reduce long-term morbidity and mortality. This research should include stratifying analyses based on gender (e.g., trans male, trans female, nonbinary) and sex assigned at birth, as well as age (e.g., prepuberty, adolescence) [71]. HCPs should be advocating for inclusive care and policies (non-stigmatizing, gender affirming, and weight inclusive) in their local institutions and communities and consider how they might join their voices to regional, national, and international efforts.

## 9. Conclusions

The assessment and treatment of ED and DEBs in youth with obesity is complex and requires a holistic approach. This approach begins with identifying and understanding how one’s implicit biases impact care. Providing care from a patient and family-centered lens, which considers how the intersection of multiple stigmatized identities increases the risk for DEBs in youth with obesity may improve long-term health outcomes.

## Figures and Tables

**Table 1:** Disordered Eating Behaviors [1,21,22].

Disordered Eating Behaviors (DEBs)		
Diagnosis	ED Presentation	Subclinical Presentation
Anorexia Nervosa (AN)	Restriction of energy intake relative to requirements, leading to significantly low body weight [1]; Intense fear of gaining weight or becoming fat even though at a significantly low weight; Disturbance in the way in which one’s body weight or shape is experienced; BMI less than 85 percent of body weight expected for age and height or failure to gain weight during a growth period, leading to body weight less than 85 percent of that expected.	Refusal to maintain body weight over a minimally normal weight, and/or Intensive fear of gaining weight or becoming fat, even though underweight [21].
Atypical Anorexia Nervosa (AAN)	All criteria for AN are met, except that despite significant weight loss, the individual’s weight is within or above the normal range for BMI [1,22].	All criteria for subclinical AAN are met, except that the individual’s weight is within or above the normal range for BMI [1].
Bulimia Nervosa (BN)	Recurrent episodes of binge eating (eating a large amount of food in a discrete period of time) + loss of control during binge eating [1]; Recurrent compensatory behavior to prevent weight gain; Self-evaluation is unduly influenced by body shape and weight; Occurs at least once a week for 3 months.	Recurrent episodes ≥ 2 times weekly of binge eating; or Loss of control during binge eating and Compensatory behaviors of preventing weight gain [21].
Binge ED (BED)	Recurrent episodes of binge eating associated with 3 or more of the following: [1] (1) Eating more rapidly than normal; (2) Eating until uncomfortably full; (3) Eating large amounts of food when not physically hungry; (4) Eating alone due to embarrassment about how much one is eating; (5) Feeling disgusted with oneself, depressed, or guilty afterward and Marked distress regarding binge eating; No compensatory behaviors to prevent weight gain; Binge eating occurs on average at least once per week for 3 months.	Having an episode of binge eating < 2 times weekly [21].

## References

[1] American Psyciatric Association (2022). Diagnostic and Statistical Manual of Mental Disorders.

[2] Jebeile H., Lister N.B., Baur L.A., Garnett S.P., Paxton S.J. (2021). Eating disorder risk in adolescents with obesity. Obes. Rev. Off. J. Int. Assoc. Study Obes..

[3] Skinner A.C., Ravanbakht S.N., Skelton J.A., Perrin E.M., Armstrong S.C. (2018). Prevalence of Obesity and Severe Obesity in US Children, 1999–2016. Pediatrics.

[4] World Health Organization (2016). Report of the Commission on Ending Childhood Obesity.

[5] Skinner A.C., Perrin E.M., Moss L.A., Skelton J.A. (2015). Cardiometabolic Risks and Severity of Obesity in Children and Young Adults. New Engl. J. Med..

[6] Rankin J., Matthews L., Cobley S., Han A., Sanders R., Wiltshire H.D., Baker J.S. (2016). Psychological consequences of childhood obesity: Psychiatric comorbidity and prevention. Adolesc. Health Med. Ther..

[7] Schwimmer J.B., Burwinkle T.M., Varni J.W. (2003). Health-related quality of life of severely obese children and adolescents. JAMA.

[8] Reilly J.J., Kelly J. (2011). Long-term impact of overweight and obesity in childhood and adolescence on morbidity and premature mortality in adulthood: Systematic review. Int. J. Obes..

[9] Hornberger L.L., Lane M.A., Adolescence T.C.O., Hornberger L.L., Lane M., Breuner C.C., Alderman E.M., Grubb L.K., Powers M., Upadhya K.K. (2021). Identification and Management of Eating Disorders in Children and Adolescents. Pediatrics.

[10] Rancourt D., McCullough M.B. (2015). Overlap in Eating Disorders and Obesity in Adolescence. Curr. Diab. Rep..

[11] Duncan A.E., Ziobrowski H.N., Nicol G. (2017). The Prevalence of Past 12-Month and Lifetime DSM-IV Eating Disorders by BMI Category in US Men and Women. Eur. Eat. Disord. Rev..

[12] Rozzell K., Moon D.Y., Klimek P., Brown T., Blashill A.J. (2019). Prevalence of Eating Disorders Among US Children Aged 9 to 10 Years: Data From the Adolescent Brain Cognitive Development (ABCD) Study. JAMA Pediatr..

[13] Smink F.R., van Hoeken D., Oldehinkel A.J., Hoek H.W. (2014). Prevalence and severity of DSM-5 eating disorders in a community cohort of adolescents. Int. J. Eat. Disord..

[14] Flament M.F., Buchholz A., Henderson K., Obeid N., Maras D., Schubert N., Paterniti S., Goldfield G. (2015). Comparative distribution and validity of DSM-IV and DSM-5 diagnoses of eating disorders in adolescents from the community. Eur. Eat. Disord. Rev..

[15] Mitchison D., Mond J., Bussey K., Griffiths S., Trompeter N., Lonergan A., Pike K.M., Murray S.B., Hay P. (2020). DSM-5 full syndrome, other specified, and unspecified eating disorders in Australian adolescents: Prevalence and clinical significance. Psychol. Med..

[16] Flament M.F., Henderson K., Buchholz A., Obeid N., Nguyen H.N., Birmingham M., Goldfield G. (2015). Weight Status and DSM-5 Diagnoses of Eating Disorders in Adolescents From the Community. J. Am. Acad. Child. Adolesc. Psychiatry.

[17] Mahr F., Farahmand P., Bixler E.O., Domen R.E., Moser E.M., Nadeem T., Levine R.L., Halmi K.A. (2015). A national survey of eating disorder training. Int. J. Eat. Disord..

[18] Sonneville K.R., Lipson S.K. (2018). Disparities in eating disorder diagnosis and treatment according to weight status, race/ethnicity, socioeconomic background, and sex among college students. Int. J. Eat. Disord..

[19] Hayes J.F., Fitzsimmons-Craft E.E., Karam A.M., Jakubiak J., Brown M.L., Wilfley D.E. (2018). Disordered Eating Attitudes and Behaviors in Youth with Overweight and Obesity: Implications for Treatment. Curr. Obes. Rep..

[20] Lewinsohn P.M., Striegel-Moore R.H., Seeley J.R. (2000). Epidemiology and natural course of eating disorders in young women from adolescence to young adulthood. J. Am. Acad. Child. Adolesc. Psychiatry.

[21] Touchette E., Henegar A., Godart N.T., Pryor L., Falissard B., Tremblay R.E., Côté S.M. (2011). Subclinical eating disorders and their comorbidity with mood and anxiety disorders in adolescent girls. Psychiatry Res..

[22] Harrop E.N., Mensinger J.L., Moore M., Lindhorst T. (2021). Restrictive eating disorders in higher weight persons: A systematic review of atypical anorexia nervosa prevalence and consecutive admission literature. Int. J. Eat. Disord..

[23] Crenshaw K. (1991). Mapping the Margins: Intersectionality, Identity Politics, and Violence against Women of Color. Stanf. Law Rev..

[24] Bowleg L. (2012). The Problem With the Phrase Women and Minorities: Intersectionality—An Important Theoretical Framework for Public Health. Am. J. Public Health.

[25] Grollman E.A. (2014). Multiple disadvantaged statuses and health: The role of multiple forms of discrimination. J. Health Soc. Behav..

[26] Grollman E.A. (2012). Multiple forms of perceived discrimination and health among adolescents and young adults. J. Health Soc. Behav..

[27] Hankivsky O. (2012). Women’s health, men’s health, and gender and health: Implications of intersectionality. Soc. Sci. Med..

[28] Himmelstein M.S., Puhl R.M., Quinn D.M. (2017). Intersectionality: An Understudied Framework for Addressing Weight Stigma. Am. J. Prev. Med..

[29] Pearson C.M., Miller J., Ackard D.M., Loth K.A., Wall M.M., Haynos A.F., Neumark-Sztainer D. (2017). Stability and change in patterns of eating disorder symptoms from adolescence to young adulthood. Int. J. Eat. Disord..

[30] Rubino F., Puhl R.M., Cummings D.E., Eckel R.H., Ryan D.H., Mechanick J.I., Nadglowski J., Ramos Salas X., Schauer P.R., Twenefour D. (2020). Joint international consensus statement for ending stigma of obesity. Nat. Med..

[31] Brylinsky J.A., Moore J.C. (1994). The identification of body build stereotypes in young children. J. Res. Personal..

[32] Roberts K.J., Polfuss M.L. (2022). Weight stigma in children and adolescents: Recommendations for practice and policy. Nursing.

[33] Puhl R.M., Lessard L.M. (2020). Weight Stigma in Youth: Prevalence, Consequences, and Considerations for Clinical Practice. Curr. Obes. Rep..

[34] Schvey N.A., Marwitz S.E., Mi S.J., Galescu O.A., Broadney M.M., Young-Hyman D., Brady S.M., Reynolds J.C., Tanofsky-Kraff M., Yanovski S.Z. (2019). Weight-based teasing is associated with gain in BMI and fat mass among children and adolescents at-risk for obesity: A longitudinal study. Pediatr. Obes..

[35] Bucchianeri M.M., Gower A.L., McMorris B.J., Eisenberg M.E. (2016). Youth experiences with multiple types of prejudice-based harassment. J. Adolesc..

[36] Puhl R.M., Wall M.M., Chen C., Bryn Austin S., Eisenberg M.E., Neumark-Sztainer D. (2017). Experiences of weight teasing in adolescence and weight-related outcomes in adulthood: A 15-year longitudinal study. Prev. Med..

[37] Thompson I., Hong J.S., Lee J.M., Prys N.A., Morgan J.T., Udo-Inyang I. (2020). A review of the empirical research on weight-based bullying and peer victimisation published between 2006 and 2016. Educ. Rev..

[38] Salmon S., Turner S., Taillieu T., Fortier J., Afifi T.O. (2018). Bullying victimization experiences among middle and high school adolescents: Traditional bullying, discriminatory harassment, and cybervictimization. J. Adolesc..

[39] Golaszewski N.M., Pasch K.E., Fernandez A., Poulos N.S., Batanova M., Loukas A. (2018). Perceived Weight Discrimination and School Connectedness Among Youth: Does Teacher Support Play a Protective Role?. J. Sch. Health.

[40] Juvonen J., Lessard L.M., Schacter H.L., Suchilt L. (2017). Emotional Implications of Weight Stigma Across Middle School: The Role of Weight-Based Peer Discrimination. J. Clin. Child Adolesc. Psychol..

[41] Morales D.X., Prieto N., Grineski S.E., Collins T.W. (2019). Race/Ethnicity, Obesity, and the Risk of Being Verbally Bullied: A National Multilevel Study. J. Racial. Ethn. Health Disparities.

[42] Lian Q., Su Q., Li R., Elgar F.J., Liu Z., Zheng D. (2018). The association between chronic bullying victimization with weight status and body self-image: A cross-national study in 39 countries. PeerJ.

[43] Koyanagi A., Veronese N., Vancampfort D., Stickley A., Jackson S.E., Oh H., Shin J.I., Haro J.M., Stubbs B., Smith L. (2020). Association of bullying victimization with overweight and obesity among adolescents from 41 low- and middle-income countries. Pediatr. Obes..

[44] Puhl R.M., Luedicke J., DePierre J.A. (2013). Parental Concerns about Weight-Based Victimization in Youth. Child. Obes..

[45] Puhl R.M., Latner J.D., O’Brien K., Luedicke J., Forhan M., Danielsdottir S. (2016). Cross-national perspectives about weight-based bullying in youth: Nature, extent and remedies. Pediatr. Obes..

[46] Pont S.J., Puhl R., Cook S.R., Slusser W. (2017). Stigma Experienced by Children and Adolescents With Obesity. Pediatrics.

[47] Bradshaw C.P., Waasdorp T.E., O’Brennan L.M., Gulemetova M. (2013). Teachers’ and Education Support Professionals’ Perspectives on Bullying and Prevention: Findings From a National Education Association Study. School. Psych. Rev..

[48] Puhl R.M., Himmelstein M.S., Pearl R.L. (2020). Weight stigma as a psychosocial contributor to obesity. Am. Psychol..

[49] Himmelstein M.S., Puhl R.M., Watson R.J. (2019). Weight-based victimization, eating behaviors, and weight-related health in Sexual and Gender Minority Adolescents. Appetite.

[50] Himmelstein M.S., Puhl R.M. (2019). Weight-based victimization from friends and family: Implications for how adolescents cope with weight stigma. Pediatr. Obes..

[51] Vartanian L.R., Porter A.M. (2016). Weight stigma and eating behavior: A review of the literature. Appetite.

[52] Pearl R.L., Wadden T.A., Jakicic J.M. (2021). Is weight stigma associated with physical activity? A systematic review. Obesity.

[53] Lydecker J.A., Grilo C.M. (2017). Does your child’s weight influence how you judge yourself as a parent? A cross-sectional study to define and examine parental overvaluation of weight/shape. Prev. Med..

[54] Roberts K.J., Polfuss M.L., Marston E.C., Davis R.L. (2021). Experiences of weight stigma in adolescents with severe obesity and their families. J. Adv. Nurs..

[55] Nutter S., Ireland A., Alberga A.S., Brun I., Lefebvre D., Hayden K.A., Russell-Mayhew S. (2019). Weight Bias in Educational Settings: A Systematic Review. Curr. Obes. Rep..

[56] Eisenberg M.E., Carlson-McGuire A., Gollust S.E., Neumark-Sztainer D. (2015). A content analysis of weight stigmatization in popular television programming for adolescents. Int. J. Eat. Disord..

[57] Clark O., Lee M.M., Jingree M.L., O’Dwyer E., Yue Y., Marrero A., Tamez M., Bhupathiraju S.N., Mattei J. (2021). Weight Stigma and Social Media: Evidence and Public Health Solutions. Front. Nutr..

[58] Alberga A.S., Pickering B.J., Alix Hayden K., Ball G.D., Edwards A., Jelinski S., Nutter S., Oddie S., Sharma A.M., Russell-Mayhew S. (2016). Weight bias reduction in health professionals: A systematic review. Clin. Obes..

[59] Pearl R.L., Puhl R.M. (2018). Weight bias internalization and health: A systematic review. Obes. Rev..

[60] Alberga A.S., Edache I.Y., Forhan M., Russell-Mayhew S. (2019). Weight bias and health care utilization: A scoping review. Prim. Health Care Res. Dev..

[61] Phelan S.M., Burgess D.J., Yeazel M.W., Hellerstedt W.L., Griffin J.M., van Ryn M. (2015). Impact of weight bias and stigma on quality of care and outcomes for patients with obesity. Obes. Rev..

[62] Jachyra P., Anagnostou E., Knibbe T.J., Petta C., Cosgrove S., Chen L., Capano L., Moltisanti L., McPherson A.C. (2018). Weighty Conversations: Caregivers’, Children’s, and Clinicians’ Perspectives and Experiences of Discussing Weight-Related Topics in Healthcare Consultations. Autism Res..

[63] Nagata J.M., Garber A.K., Tabler J.L., Murray S.B., Bibbins-Domingo K. (2018). Prevalence and Correlates of Disordered Eating Behaviors Among Young Adults with Overweight or Obesity. J. Gen. Intern. Med..

[64] Watson R.J., Adjei J., Saewyc E., Homma Y., Goodenow C. (2017). Trends and disparities in disordered eating among heterosexual and sexual minority adolescents. Int. J. Eat. Disord..

[65] Watson R.J., VanKim N.A., Rose H.A., Porta C.M., Gahagan J., Eisenberg M.E. (2018). Unhealthy weight control behaviors among youth: Sex of sexual partner is linked to important differences. Eat. Disord..

[66] Institute of Medicine (2011). The Health of Lesbian, Gay, Bisexual, and Transgender People: Building a Foundation for Better Understanding.

[67] Austin S.B., Ziyadeh N.J., Corliss H.L., Haines J., Rockett H.R., Wypij D., Field A.E. (2009). Sexual orientation disparities in weight status in adolescence: Findings from a prospective study. Obesity.

[68] Katz-Wise S.L., Blood E.A., Milliren C.E., Calzo J.P., Richmond T.K., Gooding H.C., Austin S.B. (2014). Sexual orientation disparities in BMI among U.S. adolescents and young adults in three race/ethnicity groups. J. Obes..

[69] Austin S.B., Nelson L.A., Birkett M.A., Calzo J.P., Everett B. (2013). Eating disorder symptoms and obesity at the intersections of gender, ethnicity, and sexual orientation in US high school students. Am. J. Public Health.

[70] Hadland S.E., Austin S.B., Goodenow C.S., Calzo J.P. (2014). Weight misperception and unhealthy weight control behaviors among sexual minorities in the general adolescent population. J. Adolesc. Health.

[71] Coelho J.S., Suen J., Clark B.A., Marshall S.K., Geller J., Lam P.Y. (2019). Eating Disorder Diagnoses and Symptom Presentation in Transgender Youth: A Scoping Review. Curr. Psychiatry Rep..

[72] Diemer E.W., White Hughto J.M., Gordon A.R., Guss C., Austin S.B., Reisner S.L. (2018). Beyond the Binary: Differences in Eating Disorder Prevalence by Gender Identity in a Transgender Sample. Transgend Health.

[73] McClain Z., Peebles R. (2016). Body Image and Eating Disorders Among Lesbian, Gay, Bisexual, and Transgender Youth. Pediatr. Clin. North Am..

[74] Connolly M.D., Zervos M.J., Barone C.J., Johnson C.C., Joseph C.L. (2016). The Mental Health of Transgender Youth: Advances in Understanding. J. Adolesc. Health.

[75] Diemer E.W., Grant J.D., Munn-Chernoff M.A., Patterson D.A., Duncan A.E. (2015). Gender Identity, Sexual Orientation, and Eating-Related Pathology in a National Sample of College Students. J. Adolesc. Health.

[76] Paine E.A. (2021). “Fat broken arm syndrome”: Negotiating risk, stigma, and weight bias in LGBTQ healthcare. Soc. Sci. Med..

[77] Williams D.R., Chaves E., Greenwood N.E., Kushner J., Chelvakumar G., Swaringen S.E., Leibowitz S.F. (2022). Care of Gender Diverse Youth with Obesity. Curr. Obes. Rep..

[78] van den Berg P., Neumark-Sztainer D., Eisenberg M.E., Haines J. (2008). Racial/ethnic differences in weight-related teasing in adolescents. Obesity.

[79] Hooper L., Puhl R., Eisenberg M.E., Reicks M., Neumark-Sztainer D. (2022). How is weight teasing cross-sectionally and longitudinally associated with health behaviors and weight status among ethnically/racially and socioeconomically diverse young people?. Int. J. Behav. Nutr. Phys. Act..

[80] Gordon K.H., Brattole M.M., Wingate L.R., Joiner T.E. (2006). The impact of client race on clinician detection of eating disorders. Behav. Ther..

[81] Panza E., Fehling K.B., Pantalone D.W., Dodson S., Selby E.A. (2021). Multiply marginalized: Linking minority stress due to sexual orientation, gender, and weight to dysregulated eating among sexual minority women of higher body weight. Psychol. Sex Orientat. Gend. Divers.

[82] Maguen S., Hebenstreit C., Li Y., Dinh J.V., Donalson R., Dalton S., Rubin E., Masheb R. (2018). Screen for Disordered Eating: Improving the accuracy of eating disorder screening in primary care. Gen Hosp. Psychiatry.

[83] Morgan J.F., Reid F., Lacey J.H. (2000). The SCOFF questionnaire: A new screening tool for eating disorders. West J. Med..

[84] Krabbenborg M.A., Danner U.N., Larsen J.K., van der Veer N., van Elburg A.A., de Ridder D.T., Evers C., Stice E., Engels R.C. (2012). The Eating Disorder Diagnostic Scale: Psychometric features within a clinical population and a cut-off point to differentiate clinical patients from healthy controls. Eur. Eat Disord. Rev..

[85] Stice E., Telch C.F., Rizvi S.L. (2000). Development and validation of the Eating Disorder Diagnostic Scale: A brief self-report measure of anorexia, bulimia, and binge-eating disorder. Psychol. Assess.

[86] Puhl R.M., Himmelstein M.S. (2018). Adolescent preferences for weight terminology used by health care providers. Pediatr. Obes..

[87] Obesity Action Coalition (2021). People-First Language for Obesity. https://www.obesityaction.org/wp-content/uploads/1033162_FirstPersonOne-Pager01_041921.pdf.

[88] Mason P. (2019). Health Behavior Change: A Guide for Practicioners.

